# Application of Anderson model to analyze the influencing factors of lung function test behavior in middle-aged and elderly people in China

**DOI:** 10.1016/j.dialog.2023.100138

**Published:** 2023-05-18

**Authors:** Huiwen Jiang, Bojunhao Feng, Yujia Wang, Pu Ge, Ke Lv, Xinying Sun, Yibo Wu

**Affiliations:** a School of Health Administration, Harbin Medical University, Harbin 150081, China; b School of Medicine, Macau University of Science and Technology, Macao 999078, China; c School of Humanities and Social Sciences, Harbin Medical University, Harbin 150081, China; d Institute of Chinese medical sciences, University of Macau, Macau 999078, China; e School of Public Health, China Medical University, Shenyang 110122, China; f School of Public Health, Peking University, Beijing 100191, China

**Keywords:** Middle-aged and aged people, Lung function test, Anderson model, Influence factor

## Abstract

**Introduction:**

Lung function tests are valuable in assessing respiratory health and disease, and the Healthy China Initiative clearly states that people over 40 years of age should have a lung function test once a year. To explore the influence of propensity factors, ability factors, and need factors on lung function detection behaviors of middle-aged and elderly Chinese, the following studies are conducted.

**Method:**

A questionnaire was designed using Anderson's model, and multi-stage sampling was used to conduct a nationwide questionnaire survey based on geographical subdivisions and population distribution. Frequency and percentages were used for descriptive statistical analysis of lung function testing among middle-aged and elderly people in China, and chi-square tests and binary logistic regression analyses were used to investigate the factors influencing lung function testing behavior among middle-aged and elderly people in China.

**Result:**

A total of 404 study participants were included in this study. Education level (relative to primary school and below, middle school and high school and secondary school OR = 2.652, *P* = 0.018; college and above OR = 4.566, *P* = 0.002), mode of health care affordability (relative to those who paid for the test, non-payers OR = 2.205, *P* = 0.004), dimensions of the European Five Dimensional Health Scale (mobility OR = 4.571, *P* = 0.006; pain or discomfort OR = 0.397, *P* = 0.003; anxiety or depression OR = 0.511, *P* = 0.028), and self-efficacy (medium group 0R = 0.294, *P* < 0.001; low group OR = 0.162, *P* = 0.003) had a significant impact on lung function testing behavior in our middle-aged and older adults.

**Conclusion:**

This study found that there is still room for improvement in the participation of middle-aged and elderly people in lung function testing. Among the propensity factors, the factor that affects the rate of lung function tests is the highest degree of education, which determines the degree of patients' attention to lung function tests. Among the need factors, the factors affecting the rate of lung function detection are the physical conditions of middle-aged and elderly people, and those with poor physical conditions need medical detection. Among the ability factors, the factor that affects the rate of lung function tests is the way of bearing medical expenses, and economic status is the key factor that determines whether patients can accept lung function tests.

## 1 Introduction

Lung function tests are used at the patient level for diagnosis and monitoring, as well as to assess population trends in respiratory disease over time [1]. It is valuable for the assessment of respiratory function, the diagnosis and treatment of respiratory disease, and the prognosis of outcomes [2]. Pulmonary function tests have become an indispensable tool in the clinical assessment of respiratory health and disease [3], mainly for the diagnosis of obstructive, restrictive, or mixed ventilation defects such as chronic obstructive pulmonary disease (COPD), interstitial lung disease, and asthma [4]. The importance of spirometry for the diagnosis, treatment, and assessment of disease has been noted in the Global Initiative for the Control of Chronic Obstructive Lung Disease (GOLD) [5], the Global Initiative for the Control of Bronchial Asthma (GINA) [6], and the Expert Consensus on the Management of Bronchiectasis in China [7], but is limited by the availability of spirometry in low- and middle-income countries (including spirometry methods and spirometry quality assurance) [[8], [9]], and low public awareness and weak awareness of the test, its use for clinical applications is poor. For example, chronic respiratory diseases represented by COPD have a serious impact on patients' health due to their long duration, recurrent attacks, and severe effects on multiple organs throughout the body [10]. In 2017 COPD has become the 3rd leading cause of death from disease in China [11], and the prevalence has shown an increasing trend in the last 15 years [12], seriously affecting the quality of life of middle-aged and elderly patients. The quality of life of asthmatic patients is also largely dependent on lung function status and fully emphasizes the importance of asthma treatment [13]. These suggest that we need to increase lung function testing for early intervention and treatment of COPD and asthma to improve their quality of life. At the same time, there is a negative correlation between pulse pressure difference and lung function in the middle-aged and elderly population [14], so performing lung function tests can lead to early treatment and further control of pulse pressure difference. In recent years, with the accelerated aging of the population and the increasing incidence of respiratory diseases in China, lung function tests have been more frequently used in healthcare and other fields [15]. To reduce the number of neglected diseases due to insufficient lung function detection, it is therefore important to increase efforts to study the factors that affect pulmonary function testing and thus raise awareness of pulmonary function testing.

The respiratory system increases with age and gradually declines. With the accelerated aging process of the Chinese population, aging and the change of lung function in the middle and elderly have been paid more and more attention [16]. And it is clearly stated in the Health China Initiative that people over 40 years of age should undergo lung function testing once a year, so the study chose people over 40 years of age as the study population.

In the course of studying the factors influencing previous spirometry tests, relevant studies were found to focus on the factors influencing spirometry and the effect of spirometry on specific diseases. Li Jing and Wang Huanxin et al. found that the older the female, the older the age and the lower the education level, the lower the exertional spirometry and 1s exertional expiratory volume [17]. There is also a need to reduce indoor air pollution, stay away from traffic arteries and reduce passive smoking, thus avoiding impaired lung function in the population. Wen Yajun concluded that age, gender, education level, respiratory rate, and HAMA score were all independent influencing factors in the measurement of lung function [15]. Kang Zhen and Liu Xiaobo et al. found that atmospheric PM2.5 concentrations in Harbin affected the reduction of lung function indices in school-aged children [18]. To further explore the factors influencing lung function testing, this paper uses Anderson's model to explore the factors influencing lung function testing behavior and describe the health status of middle-aged and elderly people in China.

## 2 Data and methods

### 2.1 Source of information

The data were collected using a multi-stage sampling method, based on geographical divisions and population distribution. 2–3 provinces (autonomous regions and municipalities directly under the central government) were selected from each of the 7 administrative regions of East China, South China, North China, Central China, Southwest China, Northeast China, and Northwest China using the random number table method, and 2 cities were selected from each of the selected provinces using the random number table method, skipping this step if they were municipalities directly under the central government. In other words, Shandong Province (Jinan, Jining) and Jiangsu Province (Nanjing, Wuxi) in East China; Guangdong Province (Guangzhou, Shenzhen) and Hainan Province (Haikou, Sanya) in South China; Beijing, Inner Mongolia Autonomous Region (Hohhot, Baotou) and Shanxi Province (Taiyuan, Jinzhong) in North China; Henan Province (Zhengzhou, Pingdingshan) and Hunan Province (Changsha, Xiangtan) in Central China; Sichuan Province (Chengdu, Luzhou) and Chongqing in southwest China; Liaoning Province (Shenyang, Jinzhou) and Heilongjiang Province (Harbin, Jiamusi) in northeast China; and Shaanxi Province (Xi'an, Ankang) and Xinjiang Uyghur Autonomous Region (Urumqi, Karamay) in northwest China. Quota sampling was also conducted for the population drawn (quota attributes were gender, urban-rural distribution, etc.) so that the gender and urban-rural distribution of the obtained sample matched the population characteristics.

The inclusion criteria were: (i) nationality of the People's Republic of China; (ii) permanent residence in China (time spent away from home ≤ 1 month per year); (iii) voluntary participation in the study and completion of an informed consent form; (iv) ability to complete the online questionnaire on their own or with the help of an investigator; (v) understanding of the meaning expressed in each entry of the questionnaire(Their basic abilities were evaluated by investigators during one-on-one interviews). The exclusion criteria were: (i) those who were confused or mentally ill; (ii) those who were participating in other similar research projects; and (iii) those who were unwilling to cooperate. The main method of determining whether a person is mentally abnormal is through a combination of self-reporting and systematic (some community health centers have records of this). In this part of the survey, there are usually staff in the community (neighborhood committee or health center staff) who are more knowledgeable about their community.

The survey was conducted through a large public health academic network covering several provinces in China. In each province, researchers from the School of Public Health or related departments contacted several local community health centers and neighborhood committees that were willing to cooperate with the survey to establish local networks and sites. The questionnaire is an electronic questionnaire developed by Wenjuanxing. During the survey, trained local investigators to recruit a specified number of participants (based on quota design) who meet the inclusion criteria in the community where the site is located through posters, communication tools, and face-to-face conversations. The survey subjects answered by clicking the link and obtaining their informed consent during the survey. The questionnaire information filled in by the participants would be automatically collected to the server in the background.

The data collected was finally selected for the research part of this paper, according to the specific age requirements of the research content: limited to people over 40 years of age to be included in the study, and then data with incomplete content and obvious logical problems (For example, too high or too low in height and weight, multiple data duplicates) were excluded. 428 questionnaires were received, with 404 valid questionnaires and a valid return rate of 94 %.

### 2.2 The Anderson model

**Fig. 1:**
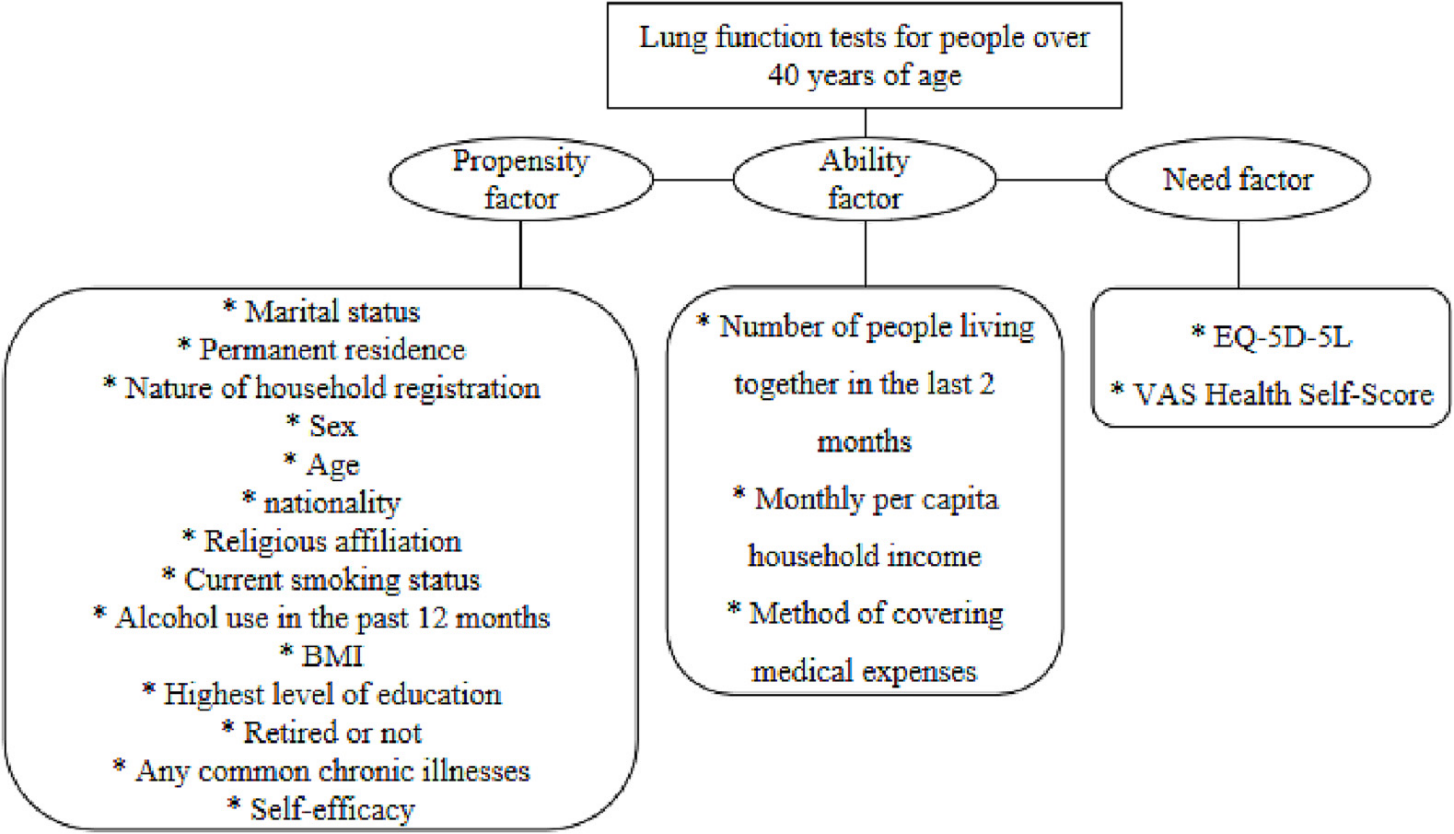
The theoretical model of this paper.

The Anderson Model is known as the Health Service Utilization Model or Health Care Utilization Behavior Model [[19], [20]]. The Anderson Model was created by the American scholar Ronald Max Anderson in 1968, so it is referred to as the “Anderson Model”, and was first used to analyze the factors influencing the Utilization of health services at home [21]. Anderson's model is widely used in health needs-oriented research to better reflect individual behavioral intentions regarding outcome variables [22], and it classifies behavioral influences into three categories: propensity, ability, and need factors [23]. Based on the purpose of the study and the accessibility of the variable information, certain adjustments were made based on Anderson's behavioral model, and the details of the adjusted model are shown in Fig. 1.

A systematic analysis of the strengths and weaknesses of the Anderson Model using SWOT analysis, and the opportunities and risks of the current situation, to explore the feasibility of promoting the Anderson Model. Strengths: The Anderson Model is a highly authoritative research model in the field of health services research [24]. It can use a systems science perspective to analyze the utilization of health services and identify the factors that influence it, thereby helping policymakers to use policy instruments to improve access to health services. Disadvantages: The generalization and classification of certain factors may be controversial. Variables that are relatively abstract in definition may be more arbitrary in the selection of indicators. Opportunities: Such studies fit well with the Anderson model. At the same time, the introduction of the Anderson model into the study allows for a more standardized study and makes it easier to compare the results obtained with similar international studies. Threat: Awareness of similar studies in China using the Anderson model is weak.

## 3 Research tools

### 3.1 Dependent variable

Pulmonary function testing status, which was a single-choice question; the question was: perform pulmonary function testing once a year; there were six options: do not intend to adopt the behavior, intend to adopt the behavior but have not yet decided when to start, decide to adopt the behavior soon or immediately, start trying to adopt the behavior, have adopted the behavior but have not adhered to it for a long time, and have adhered to the behavior for a long time. In this study, the first three options were combined as not having adopted the behavior, and the last three options were combined as having adopted the behavior at least once, based on whether or not the lung function testing behavior had been adopted in action. A value of 0 was assigned to not having taken the behavior and 1 to having taken the behavior at least once.

### 3.2 Independent variables

#### 3.2.1 Composition of propensity factors

The study of propensity factors consisted mainly of basic information on demographic characteristics and household characteristics, including marital status, place of permanent residence, nature of household registration, gender, age, ethnicity, religion, current smoking status, whether or not they had consumed alcohol in the past 12 months, BMI, highest educational level, whether or not they were retired, whether or not they had common chronic diseases (hypertension, stroke, coronary heart disease, asthma, chronic obstructive pulmonary disease, diabetes, malignancy, mood disorders (anxiety disorders, depression, etc.)) and self-efficacy.

Self-efficacy is a subjective assessment of an individual's ability to perform a certain task, and the outcome of this assessment will have a direct impact on a person's motivation to act, so self-efficacy can have an impact on healthcare decisions to some extent. The new General Self-Efficacy Scale (NGSES) consists of eight items with five options: totally disagree; disagree; both agree and disagree; agree; and totally agree. In this paper, self-efficacy was scored by referring to the General Self-Efficacy Scale (GSS) [25], firstly, each option was assigned a score: totally disagree = 1; disagree = 2; neither agree nor disagree = 3; agree = 4; totally agree = 5. The higher the total score, the higher the self-efficacy of the respondents [25]. The reliability of the scale was good, with Cronbach's α = 0.872 [26].

#### 3.2.2 Composition of ability factors

The study of the ability factor consists mainly of the number of people living with the family in the last 2 months, the monthly per capita income of the family, and how medical expenses are covered.

#### 3.2.3 Composition of the need factors

The study of the need factor consists mainly of the European Five Dimensions Health Scale (EQ-5D-5L) and the Health Effect Value Visual Analogue Score (VAS).

The European Five-Dimensional Health Scale (EQ-5D-5L), as a measure of the health status of patients or healthy people, is the most widely used health-related quality of life (HRQOL) measurement tool [27], which studies people's survival and health of life through universal scales. The reliability of the Chinese version of the European Five Dimensions Health Scale (EQ-5D-5L) is good, with Cronbach's α = 0.624 and a KMO test value of 0.685 [28]. The European Five Dimensions Health Scale (EQ-5D-5L) consists of questions on five dimensions: mobility, self-care, daily activities, pain or discomfort, and anxiety or depression, with each dimension consisting of five options: no problems, a little problem, moderate problems, serious problems, and unable to perform/have very serious problems. In this paper, having a little problem, having a moderate problem, having a serious problem, and being unable to proceed/having a very serious problem are combined into having a problem, and the final combined result is no problem and having a problem for two targets.

The Health Effect Value Visual Analogue Score (EQ-5D-VAS) reflects people's health status by rating how well they are doing. The VAS is a scale with numbers from 0 to 100, with 100 representing the best health one can imagine and 0 representing the worst health one can imagine [23]. In this paper, the VAS score is divided into 3 levels, with a VAS health score of 60 or less being poor, 60 to 79 being fair and 80 or more being good [23]. The retest reliability was 0.99, and Elton et al. showed that the parallel validity of VAS and MPQ ranged from 0.60 to 0.63 [29], with good reliability and validity.

## 4 Quality control

Questionnaires were conducted by uniformly trained enumerators following the implementation plan, and the cooperation of the survey respondents was fully obtained before the survey. The questionnaire must be answered by the respondents thinking independently to ensure the authenticity of the survey. If the survey respondents had language barriers or low literacy levels, appropriate explanations might be given, but the explanations should be faithful to the original meaning. Quality control officers carefully checked the information collected to exclude questionnaires with partially scored blank items and exclude questionnaires with inconsistent logic checks.

## 5 Data processing methods

Data were analyzed using SPSS 26.0 statistical software. Firstly, the general characteristics of the study subjects were used such as frequencies and percentages to describe the subjects' demographic characteristics, family characteristics, EQ-5D-5L health status, VAS scores, and self-efficacy in general. Next, based on the Anderson Behavioural Model [23](Fig. 1) was conducted for univariate analysis, and their rates were compared with the composition ratios using chi-square tests. Finally, stepwise binary logistic regression analysis was conducted to examine the influence of various factors on lung function testing.

In this paper, subjects were selected, grouped, and assigned values for whether they had adopted the act of lung function testing as the dependent variable. Sociodemographic characteristics, and family characteristics as covariates.EQ-5D-5L health status, VAS score, and self-efficacy as independent variables (Table 1). The test was α = 0.05.

**Table 1:** Variable assignments for univariate and multivariate analyses.

	Variable	Assignment
Propensity factor	Marital status	Married = 0; Other = 1
	Place of permanent residence	Town = 0; Village = 1
	Nature of household	Non-agricultural = 0; Agricultural = 1
	Gender	Male = 0; Female = 1
	Age	41–50 = 0; ≥51 = 1
	Ethnicity	Han Chinese = 0; Other ethnic groups = 1
	Religious belief	No religious affiliation = 0; Religious affiliation = 1
	Smoking status	No history of smoking = 0; History of smoking = 1
	Have you consumed alcohol in the last 12 months	Never drink = 0; Drink = 1
	BMI(kg/m^2^)	<18.5 = 0; 18.5–23.9 = 1; ≥24 = 2
	Highest level of education	Primary school and below = 0; Middle school and high school, secondary school = 1; College and above = 2
	Retired or not	No = 0; Yes = 1
	Any common chronic diseases (e.g. hypertension, diabetes, etc.)	No = 0; Yes = 1
	Self-Efficacy(NGSES)	>30(high grouping) = 0; 20–30 (medium grouping) = 1; <20 (low grouping) = 2 (completely disagree = 1; disagree = 2; both agree and disagree = 3; agree = 4; completely agree = 5)
Competence factor	Number of people living together in the last 2 months	0–1 people = 0; 2–3 people = 1; 4–8 persons = 2
	Monthly per capita household income (RMB)	≤3000 = 0; 3001–6000 = 1; ≥6001 = 2
	How medical costs are covered	Self-funded = 0; Non-self-funded = 1
Need factor	EQ-5D-5L	
	Mobility	No problem = 0; Problem = 1
	Self-care	No problem = 0; Problem = 1
	Daily activity	No problem = 0; Problem = 1
	Pain or discomfort	No problem = 0; Problem = 1
	Anxiety or depression	No problem = 0; Problem = 1
	VAS	80–100 points (high grouping) = 0; 61–79 points (medium grouping) = 1; 0–60 points (low grouping) = 2
Dependent variable	Pulmonary Function Test Status	Has not committed the act = 0; has committed the act at least one time = 1

## 6 Results

### 6.1 Descriptive statistics

#### 6.1.1 Descriptive statistics of propensity factors

A total of 404 respondents were included in this paper; 57.2 % were aged 41–50; the majority of marital status were married (90.1 %); 75.7 % were urban in their place of residence; 67.8 % were non-agricultural; the gender ratio was the same for both sexes (50 %); the majority were Han Chinese (94.6 %) and had no religious beliefs (90.1 %); the majority had no The majority of respondents had no history of smoking (70.8 %); 53.7 % had never consumed alcohol; 48.3 % had a BMI of 18.5–23.9; 51.2 % had an education level of college or above (Table 2); 70.8 % were not retired; 57.7 % had no common chronic diseases; 62.1 % were in the high self-efficacy group; 31.7 % were in the medium group; and 6.2 % were in the low group. The high self-efficacy group accounted for 62.1 %; the medium group accounted for 31.7 %; the low group accounted for 6.2 % (Table 2).

**Table 2:** Demographic characteristics and results of univariate analysis of lung function tests in middle-aged and elderly people (*n* = 404).

Indicator	Group	Number of people	Composition ratio (%)	Number of people tested	Detection rate(%)	χ^2^	*P*
Marital status	Married	364	90.1	206	56.6	0.636	0.425
	Other	40	9.9	20	50.0		
Number of people living together in the last 2 months	0–1 person	181	44.8	100	55.2	2.233	0.327
	2–3 person	181	44.8	98	54.1		
	4–8 person	42	10.4	28	66.7		
Place of permanent residence	Town	306	75.7	174	56.9	0.435	0.509
	Village	98	24.3	52	53.1		
Nature of household	Non-agricultural	274	67.8	161	58.8	2.745	0.098
	Agriculture	130	32.2	65	50.0		
Monthly per capita household income (yuan)	≤3000	120	29.7	56	46.7	6.780	**0.034**
	3001–6000	134	33.2	84	62.7		
	≥6001	150	37.1	86	57.3		
Gender	Male	202	50.0	115	56.9	0.161	0.689
	Female	202	50.0	111	55.0		
Age	41–50	231	57.2	139	60.2	3.921	**0.048**
	≥51	173	42.8	87	50.3		
Ethnicity	Han Chinese	382	94.6	212	55.5	0.559	0.455
	Other nationalities	22	5.4	14	63.6		
Religious belief	No religious affiliation	364	90.1	210	57.7	4.577	**0.032**
	Religious affiliation	40	9.9	16	40.0		
Smoking status	No history of smoking	286	70.8	161	56.3	0.050	0.824
	History of smoking	118	29.2	65	55.1		
Have you consumed alcohol in the last 12 months	Never drink alcohol	217	53.7	126	58.1	0.858	0.354
	Drinking	187	46.3	100	53.5		
BMI(kg/m^2^)	<18.5	18	4.4	10	55.6	1.103	0.576
	18.5–23.9	195	48.3	104	53.3		
	≥24	191	47.3	112	58.6		
Highest level of education	Primary school and below	57	14.1	21	36.8	13.506	**0.001**
	Junior and Senior High School, Secondary School	140	34.7	74	52.9		
	Tertiary and above	207	51.2	131	63.3		
How medical costs are covered	Self-financed	129	31.9	56	43.4	12.071	**0.001**
	Non-self-funded	275	68.1	170	61.8		
Retired or not	No	286	70.8	172	60.1	7.006	**0.008**
	Yes	118	29.2	54	45.8		
Any common chronic diseases (e.g. hypertension, diabetes, etc.)	No	233	57.7	136	58.4	1.317	0.251
	Yes	171	42.3	90	52.6		
VAS	High grouping	276	68.3	175	63.4	19.737	**<0.001**
	Medium grouping	84	20.8	34	40.5		
	Low grouping	44	10.9	17	38.6		
Self-efficacy	High grouping	251	62.1	171	68.1	41.669	**<0.001**
	Medium grouping	128	31.7	49	38.3		
	Low grouping	25	6.2	6	24.0		

#### 6.1.2 Descriptive statistics of ability factors

In terms of ability factors, the proportion of 0–1 and 2–3 persons in the same household in the last 2 months was 44.8 %; 37.1 % of the households had a per capita monthly income of ≥6001 RMB; and 68.1 % of the medical expenses were borne by non-self-paying households (Table 2).

#### 6.1.3 Descriptive statistics of need factors

The Health Effect Value Visual Analogue Score (VAS) evaluates overall health, with 80 and above being a high score (68.3 %), 61–79 being a medium score (20.8 %), and 60 and below being a low score (10.9 %) (Table 2).

In the EQ-5D-5L, the highest percentage of “no problems” was for “self-care” (83.7 %) and the highest percentage of “problems” was for “Pain or discomfort” (46.3 %) (Table 3).

**Table 3:** Five-dimensional self-assessment status of the population tested for lung function.

Dimensionality	No problem				Problem				χ^2^	*P*
	Number of people	Percentage(%)	Testing number of people	Detection rate(%)	Number of people	Percentage(%)	Testing Number of people	Detection rate(%)		
Mobility	303	75.0	176	58.1	101	25.0	50	49.5	2.263	0.132
Self-care	338	83.7	194	57.4	66	16.3	32	48.5	1.779	0.182
Daily activity	321	79.5	190	59.2	83	20.5	36	43.4	6.694	**0.010**
Pain or discomfort	217	53.7	147	67.7	187	46.3	79	42.2	26.491	**<0.001**
Anxiety or depression	236	58.4	154	65.3	168	41.6	72	42.9	19.974	**<0.001**

### 6.2 Univariate analysis of lung function testing in middle-aged and older adults

Univariate analyses were conducted using whether the study participants had adopted the act of lung function testing as the dependent variable, and propensity, ability, and need factors as independent variables. The results showed that age, religion, the highest level of education, whether retired, self-efficacy, monthly per capita household income, mode of health care affordability, “daily activities” in the EQ-5D-5L, “pain or discomfort” and “anxiety or depression”, and VAS were statistically significantly different (*P* < 0.05) from each other in terms of whether the study participants chose to be tested. In contrast, the effects of marital status (*P* = 0.425), nature of household registration (*P* = 0.098), and BMI (*P* = 0.576) were not statistically significantly different (*P* > 0.05) on whether or not middle-aged and older adults were tested (Table 2, Table 3).

### 6.3 Multi-factor analysis of lung function tests in middle-aged and older adults

The results of the multifactorial analysis showed that the factors affecting lung function testing in middle-aged and older people were the highest literacy level, self-efficacy, mode of health care affordability, mobility, pain or discomfort, and anxiety or depression, with differences all statistically significant (*P* < 0.05) (Table 4).

**Table 4:** Multifactorial analysis of lung function tests in middle-aged and older adults.

Indicator	B	Standard error	Waldχ^2^	*P*	OR	OR95%C.I.	
						Lower limit	Upper limit
Highest level of education (control group = primary school and below)			9.462	**0.009**			
Junior and Senior High School, Secondary School	0.975	0.413	5.567	**0.018**	2.652	1.180	5.961
Tertiary and above	1.519	0.494	9.461	**0.002**	4.566	1.735	12.017
Method of covering medical costs (control group = self-pay)							
Non-self-funded	0.791	0.271	8.505	**0.004**	2.205	1.296	3.750
EQ-5D-5L							
Mobility (control group = no problem)							
problem	1.520	0.553	7.540	**0.006**	4.571	1.545	13.524
Self-care (control group = no problem)							
problem	1.341	0.693	3.741	0.053	3.822	0.982	14.874
Daily activity (control group = no problem)							
problem	−0.690	0.604	1.303	0.254	0.502	0.154	1.640
Pain or discomfort (control group = no problem)							
problem	−0.924	0.315	8.591	**0.003**	0.397	0.214	0.736
Anxiety or depression (control group = no problem)							
problem	−0.672	0.305	4.842	**0.028**	0.511	0.281	0.929
Self-efficacy (control group = high subgroup)			22.076	**<0.001**			
Medium grouping	−1.224	0.282	18.829	**<0.001**	0.294	0.169	0.511
Low grouping	−1.822	0.611	8.893	**0.003**	0.162	0.049	0.535
Constants	−1.191	1.013	1.384	0.239	0.304		

The rate of pulmonary function testing increased as literacy levels increased; it decreased as self-efficacy scores decreased; the rate of pulmonary function testing was 2.205 times higher for respondents who were not self-funded than for those who were self-funded in terms of their mode of health care affordability (OR = 2.205, *P* = 0.004); respondents with mobility problems had a higher rate of pulmonary function testing than those with no problems (OR = 4.571, *P* = 0.006); and 0.397 (OR = 0.397, *P* = 0.003) and 0.511 (OR = 0.511, *P* = 0.028) times lower among those with problems in pain or discomfort, anxiety or depression than among controls with no problems.

## 7 Discussion

### 7.1 Influence of propensity factors on participation in lung function testing in middle-aged and older adults

In terms of age, testing rates were higher among younger people. This may be because younger people are more able to actively obtain and understand information about respiratory health and receive education about spirometry than older people, and their awareness of active participation in spirometry is correspondingly higher. The participation rate in spirometry was higher among the more educated middle-aged and older adults. Possible reasons for this are that more educated middle-aged and older people have a greater ability to access and understand respiratory health information than less educated people, have a more comprehensive knowledge of health care, and place a higher value on early screening for respiratory disease. In terms of occupational status, the participation rate in pulmonary function testing was higher among non-retired middle-aged and older people compared to retired people. On the one hand, the overall age of the non-retired middle-aged and elderly is younger than that of the retired; on the other hand, with the national promotion of “including pulmonary function tests in routine medical examinations for people aged 40 and above”, some employers have also included pulmonary function tests in their routine medical examinations [30] This has increased the chances of non-retired people participating in lung function tests or receiving health education on the content. The above findings are similar to the fact that adults with impaired lung function are more likely to be older, less educated, and not working [31], from which it can be hypothesized that factors influencing the severity of impaired lung function are associated with factors affecting lung function testing rates.

In terms of religious affiliation, the testing rate was higher among those with no religious affiliation than those with religious affiliation. This may be because people without religious beliefs have a more scientific outlook and are less resistant to testing, whereas people with religious beliefs may be resistant to testing because of their religious beliefs.

In terms of self-efficacy levels, the higher the self-efficacy scale scores of middle-aged and older adults, the higher the participation rate in lung function testing. Possible reasons for this are that self-efficacy is an important factor in maintaining healthy behaviors or changing important behaviors, and those with higher self-efficacy tend to be more confident, have higher beliefs about their ability to pursue health [[32], [33]], and are more proactive in acquiring health knowledge and adopting relevant health behaviors. As a result, people with higher self-efficacy are more health literate in respiratory health and more likely to participate in lung function tests.

### 7.2 Ability factors were the main influencing factor on participation in lung function testing among middle-aged and elderly people

The rate of lung function testing was significantly higher among those whose medical costs were borne by non-self-payers than self-payers; in terms of monthly per capita household income, although the univariate analysis showed the highest testing rate among those with an income of RMB 3001-6000, the second highest rate among those with an income ≥ RMB 6001, and the lowest rate among those with an income ≤ RMB 3000, a follow-up analysis of those with an income of RMB 3001 A further chi-square split test between those with incomes of RMB 3001-6000 and those with incomes of ≥6001 revealed a statistically insignificant difference at *P* = 0.358, indicating that those with incomes >3000 had the same rate of lung function testing, both higher than those with incomes ≤3000. Combined with the multifactorial analysis it is clear that the real factor influencing lung function testing is how medical costs are borne, and that groups with higher incomes also tend to have better medical coverage.

### 7.3 Effect of need factors on participation in lung function tests in middle-aged and older adults

In the theoretical model developed in this paper based on Anderson's behavioral theory, the need factors include EQ-5D responses and VAS health self-ratings. The results of the multifactorial analysis showed that the need factor was an important factor influencing the participation of middle-aged and elderly people in the spirometry test.

In terms of EQ-5D responses, those with mobility problems had higher participation rates in spirometry than those with no problems; those with problems in the pain or discomfort, anxiety, or depression dimensions had lower participation rates in spirometry than those with no problems. Possible reasons for this include the fact that middle-aged and older people with mobility difficulties have more serious health problems of their own and therefore need to include a wider range of items in their health screening and are more likely to have a lung function test. On the other hand, this group requires more care in their daily lives and their participation in health screening programs is mainly arranged by their carers and is more comprehensive and complete. Middle-aged and older people who have difficulty with pain or discomfort, anxiety, or depression have relatively fewer health problems and place less emphasis on the need for and importance of a comprehensive health check. At the same time, those with lower quality of life may have lower levels of self-efficacy [34], which also leads to lower participation in lung function testing in this group.

In terms of VAS health self-ratings, lung function testing rates were in descending order for the high, medium, and low subgroups of the VAS health self-ratings. Overall, those with higher levels of health self-rating and actual health were associated with higher rates of lung function testing. Possible reasons for this include: the overall age distribution of those with a higher VAS health self-rating and actual health level is relatively young and they are more likely to take the initiative to test; also, due to self-efficacy factors, those with better health have a higher level of self-efficacy and are more likely to take the initiative to participate in lung function testing.

## 8 Conclusion

In conclusion, this study found that there is still room for improvement in the participation of lung function tests in middle-aged and elderly people. Among the tendency factors, the factor affecting the lung function test rate is the highest educational level, which determines the importance of patients to the lung function test. Among the need factors, the factors that affect the detection rate of lung function are the physical condition of middle-aged and old people, and the poor physical condition requires medical detection. Among the ability factors, the influencing factor of pulmonary function test rate is the way of medical expenses, and the economic status is the key factor to determine whether patients can accept pulmonary function test. Therefore, to increase the participation rate of pulmonary function tests among the middle-aged and elderly, the participation rate should be increased by targeting the health conditions of different groups of middle-aged and elderly people, starting with primary health care and enhancing their awareness of early diagnosis and treatment of respiratory diseases through health knowledge disseminated by health care providers such as the community [35]. At the same time, the state should also pay more attention to the lung function testing of the middle-aged and elderly, and increase investment in medical insurance and other aspects.

## 9 Strengths and limitations

After extensive reading of the literature, it was found that most of the articles studied the influencing factors of lung function, or the impact of lung function testing on specific diseases, while relatively few articles studied the influencing factors of lung function testing in middle-aged and elderly people, with some innovation in content. Secondly, Anderson's model is used throughout the text to analyze and categorize the influencing factors, exploring the influence of middle-aged and older people in China on lung function testing behavior in terms of propensity, ability, and need factors. Finally, the Anderson model is recognized as an authoritative research model in the field of health service research, which can explain and predict individual health service utilization behavior more comprehensively.

However, this study also has certain limitations. Firstly, this study is a cross-sectional survey, and the influencing factors explored cannot be inferred in a strictly causal sense, some longitudinal studies can be done in the future to explore their causal relationships; secondly, the reporting method of this paper is a patient-reported outcome, which is subjective and may have recall bias; thirdly, the sampling method of this paper is non-random, which may cause selection bias. Fourth, the Anderson model used has a large space for the selection of variables, so all variables cannot be included in it. Finally, as it is an online questionnaire, many middle-aged and elderly people were unable to participate and the sample size is small, which may not be representative of the overall situation in China.

## Declaration of Competing Interest

No existing or potential conflict of interest relevant to this article was reported.
